# Investigating Associations Between Developmental Integration and Physiological Stress

**DOI:** 10.1002/ajpa.70297

**Published:** 2026-06-23

**Authors:** B. R. Wigley, P. G. Blackwell

**Affiliations:** ^1^ College of Dentistry, University of Illinois Chicago USA; ^2^ School of Mathematical and Physical Sciences, University of Sheffield Sheffield UK

**Keywords:** developmental integration, geometric morphometrics, os coxae, stress marker

## Abstract

**Objectives:**

Integration, or patterns of correlation between structural elements, is of interest in diverse fields. Analysis is, however, generally limited to group‐level comparisons. This paper presents a novel combination of methods to quantify developmental integration (i.e., patterns of covariation which arise during growth) so that a univariate score is computed, summarizing individual integration in contrast to group‐level trends. This provides a means of investigating factors which influence variation in multi‐scale detail.

**Materials and Methods:**

Human ossa coxae from archaeological remains were assessed. After capturing os coxae shape through geometric morphometric methods, patterns of integration between modules—that is, the ilium, ischium and pubis—were summarized via individual scores calculated through a Leave‐One‐Out (LOO) approach. Osteological assessments allowed integration to be explored in relation to demographic factors and physiological stress experience during development as implied through chronologically matched linear enamel hypoplasia (LEH).

**Results:**

While skeletal sex and site of origin influenced gross os coxae shape, variation in integration appeared to be related to stress experienced during development, with individuals exhibiting matched LEH having significantly lower integration scores.

**Discussion:**

It is possible that developmental stress is associated with decreased integrated skeletal growth and, while gross shape is unaffected, the traces of this disruption can be detected through the method presented here. While analyses implied that this hypothesis must be treated cautiously, it is proposed that quantifications of integration in skeletal materials can potentially be employed to investigate developmental stress experience. Further work is needed to validate these assumptions.

## 1 Background

### 1.1 Developmental Integration

Complex structures are often separable into component parts, referred to as modules, which can be either more or less related in terms of their developmental process, evolutionary history, final form, and/or function (Adams 2016, 565; Klingenberg and Zaklan 2000, 1273; Zelditch and Swiderski 2023, 147). In highly integrated structures, trait variation in one module is interdependent with that in other modules (Bookstein et al. 2003, 168; Klingenberg and Zaklan 2000, 1273; Olson and Miller 1958; Zelditch and Swiderski 2023, 147). Patterns of integration are of interest for diverse reasons as they speak to adaptation (e.g., in developmental processes or phenotypic outcomes) and alteration in response to selective pressures and environmental factors (Collyer et al. 2015; Gόmez‐Robles and Polly 2012).

The analysis of integration has progressed greatly over the last decade. While linear measures can be examined for patterns of integration, as subtle structural patterns are often more apparent in shape than size, geometric morphometric (GM) investigations are now common (Adams 2016; Klingenberg and Zaklan 2000; Zelditch and Swiderski 2023). GM approaches permit shape variation to be isolated. Coordinate‐based Procrustean techniques allow objects to be defined with Cartesian points and aligned to remove variation due to differences in location, size, and orientation (Bookstein 1991; Bookstein 1997; Dryden and Mardia 2016). Quantification of developmental integration in Procrustes‐aligned shapes is achieved by calculating the covariance between coordinate points, then partitioning the covariance matrix into blocks which contain all the points from each module; within‐ and between‐block variation can be compared to estimate the degree of morphological integration (Adams 2016; Zelditch and Swiderski 2023). The value of GM approaches to the analysis of integration was, however, initially limited due to the dependence of summary statistics on sample size and number of coordinate points (Adams and Collyer 2016). To move beyond this obstacle, further post hoc procedures have been developed to produce standardized scores (e.g., z scores) which are comparable between samples (Adams and Collyer 2016; Conaway and Adams 2022).

While these methods quantify integration at the sample level, they do not provide individual‐specific measures that allow comparison of integration between individuals within the same sample (i.e., multi‐scale investigations are not possible). The development of individual univariate scores for other morphological patterns, such as fluctuating asymmetry (FA), has however proven to be interrogatively useful when assessing skeletal remains. For example, by exploring univariate FA scores, it has been demonstrated that groups exposed to greater physiological stress during development exhibit disruptions to normal patterns of growth, impacting diverse aspects of phenotype, including skeletal morphology (e.g., Weisensee 2013; Wigley et al. 2024). In this sense, stressors are defined as stimuli that alter normal somatic functioning (Escós et al. 2000, 331; Selye 1973). As FA results from the dysregulation of coordinated growth (e.g., between left and right sides) (Klingenberg 2015, 851–855; Van Valen 1962), it was theorized that the developmental integration of skeletal structures (i.e., the coordinated growth of bones containing multiple modules) may be similarly affected by stress and that this would be evident morphologically. Due to its developmental process and interdependent patterns of variation (see below), it was believed that the os coxae was suited to (1) exploring relationships between developmental stressors and skeletal integration, and (2) testing whether or not univariate integration scores could be useful as a research metric.

### 1.2 Os Coxae Development and Evolution

The os coxae is composed of three elements: the ilium, ischium, and pubis. A complicated developmental and evolutionary history, which includes the coordinated growth of these three modules in response to varied environmental and functional factors, implies complex patterns of covariation and provides the rationale for selection here.

**FIGURE 1:**
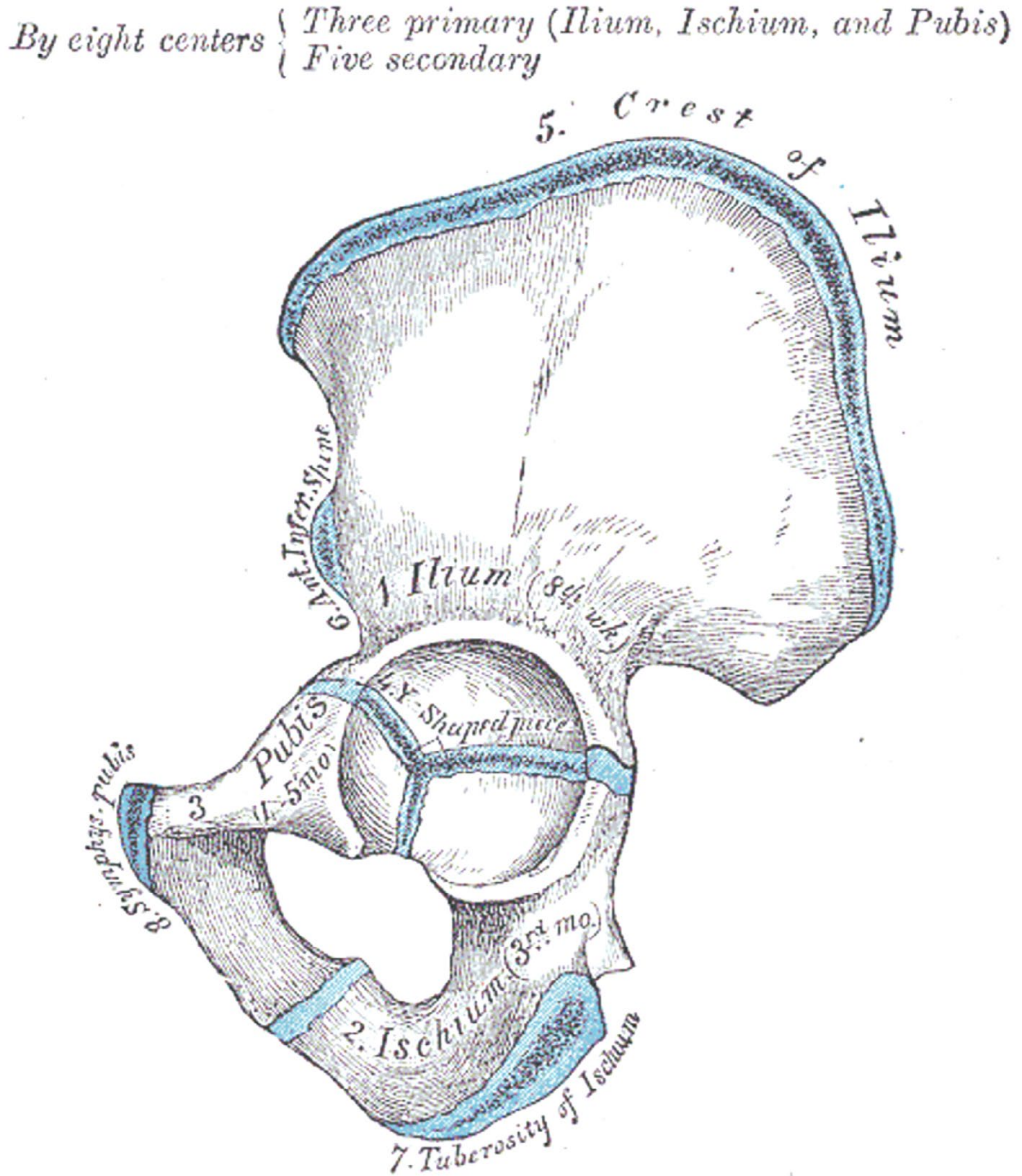
A lateral view of the left side ossa coxa illustrating the ilium, ischium and pubis. Also highlighted (light blue) are their secondary ossification centers and the connecting joints which fuse in maturity (Gray 1918, 238).

The ilium, ischium, and pubis (Figure 1) initially develop separately. Development begins in the first trimester with the condensing of cartilage cells first in the center of the embryonic ilium, followed by the pubis, and later the ischium. Ossification of each element begins in utero and, though much unossified cartilage is still present, by birth the three bones are evident (Schaefer et al. 2009, 253; Standring 2016, 1342). Before reaching maturation, central connection is provided by the triradiate cartilage, allowing the continued growth of each element throughout childhood and adolescence, with notable sex‐dependent differences in the timing and tempo of maturation (Schaefer et al. 2009, 253; Standring 2016, 1342). Fusion of the rami which connect the pubis to the other elements is variable, but precedes ossification of the triradiate cartilage and completion of the acetabulum which generally occurs in the mid‐to‐late teens (Cardoso et al. 2013; Schaefer et al. 2009, 253). Even after this, secondary centers of ossification exist, with some (such as the iliac crest) potentially persisting into the third decade of life (Schaefer et al. 2009, 229–254; White et al. 2012, 227–240). Following fusion of primary and secondary ossification centers, each bone contributes variably to different functional roles (e.g., relating to locomotion, thermoregulation, obstetrical requirements) (Candelas Gonzalez et al. 2017; Gosling et al. 2002; Kurki 2007; Ruff 1993; Warrener 2023). Due to the separation of the ilium, ischium, and pubis for key stages of development, they can be conceptualized as developmental modules. Though this is a simplification (see Discussion for limitations associated with this perspective), it provides an opportunity to investigate developmental integration in the os coxae.

From an evolutionary perspective, the morphology of the human os coxae is highly derived (e.g., Claxton et al. 2025; Fischer et al. 2021). Compared with great apes, such as the chimpanzee, the angle between the ilium and ischium is more acute. This angulation maintains a greater distance between the ischium and the femur in humans when standing, meaning the hamstring muscles have more advantageous mechanical leverages when in an erect posture, facilitating bipedal locomotion (Aiello and Dean 1990, 247; Standring 2016, 1345). Similarly, the reduced height, anterior curvature, and pronounced pillar of the human ilium, when contrasted to that of other apes, permit more efficient activation of the gluteal muscles necessary for obligate bipeds (Aiello and Dean 1990, 247; Claxton et al. 2025, 19; Standring 2016, 1344). Other morphological adaptations have evolved in the human os coxae, notably in relation to obstetric and thermoregulatory demands. There are notable sex differences in os coxae, with the female being larger and the pelvic inlet generally wider. This tendency is more evident in humans than other apes due to the increased encephalization of human fetuses and the selective pressure this places on os coxae size and shape (Candelas Gonzalez et al. 2017; Claxton et al. 2025, 20–22; Aiello and Dean 1990, 247; Fischer et al. 2021; Wells et al. 2012). The length and angulation of the iliac blades are further influenced by the need to regulate body temperature. Unsurprisingly, this varies considerably and depends on environmental conditions, the ratio of body surface area to body mass, and thoracic dimensions (Mitteroecker et al. 2021, 11; Ruff 1993). It has been proposed that the suite of selective pressures placed on the os coxae can create conflicting demands on constrained physiological resources which necessitate trade‐offs, contributing to within‐ and between‐group variation (Mitteroecker et al. 2021, 13; Pomeroy et al. 2012). In sum, it can be expected that the modules of the human os coxae exhibit highly complex patterns of covariation, potentially reflecting conflicting selective pressures resulting from environmental conditions and developmental constraints. This makes the structure ideally suited to investigations of integration.

### 1.3 Developmental Stress

Investigating physiological stress experienced during development in skeletal remains is challenging. Retrospective identification and quantification of hormonal variation, such as in the stress hormone cortisol, is subject to uncertainty and practical obstacles (e.g., ethical considerations relating to destructive analyses and access to specialized equipment) (Betts et al. 2017; Quade et al. 2020). Moreover, the remains of non‐adults whose skeletons have not fully developed represent a biased cohort of non‐survivors whose experience of stress may not accurately reflect normal trends (DeWitte and Stojanowski 2015, 416–418; Wood et al. 1992, 349). In contrast, continuous remodeling of bone throughout life may obscure or erase the traces of developmental stress in skeletally mature adults (White et al. 2012, 37). Fortunately, the dentition forms in a lengthy process beginning in utero and lasting until early adulthood. While dental enamel does not remodel in later‐life, tooth morphogenesis and amelogenesis are impacted by stress experienced during development (Guatelli‐Steinberg 2016; White et al. 2012, 107). Thus, developmental stress can be inferred through proxies such as dental fluctuating asymmetry and linear enamel hypoplasia (LEH) (e.g., Guatelli‐Steinberg 2016; Wigley et al. 2024). LEH are a particularly well‐used means of exploring stress and by evaluating bilateral LEH or matching defects across synchronously forming bands of teeth, it is possible to more confidently identify stress‐induced defects (i.e., rather than those caused by localized trauma) (Towle and Irish 2020; Primeau et al. 2015). Therefore, although factors such as dental wear and caries can obliterate LEH, heterogenous frailty complicates interpretation (see Discussion), and causality cannot be determined when relationships between LEH and other observations are detected (i.e., due to the retrospective nature of osteological analyses), LEH have been used to imply a record of stress in numerous and diverse projects (e.g., DeWitte 2018; Guatelli‐Steinberg et al. 2004).

### 1.4 Hypotheses

To explore the suppositions that (1) a relationship between os coxae integration and physiological stress experienced during development could be detected, and (2) individual univariate integration scores provide useful research metrics, two hypotheses were developed. As past research has suggested that developmental stress disrupts coordinated skeletal growth (e.g., Klingenberg 2015, 851–855; Selye 1973; Van Valen 1962), framing of these hypotheses implied directionality (but not causation).

– *Null Hypothesis:* individuals with LEH, or cohorts with a higher frequency of LEH, would not exhibit decreased os coxae integration scores
– *Alternative Hypothesis:* os coxae integration scores would be lower in individuals with LEH, or cohorts with a higher frequency of LEH

## 2 Materials

Forty‐three fully‐fused, human ossa coxae from archaeological cemetery excavations were assessed. The ossa coxae originated from two skeletal assemblages, curated by the University of Sheffield's Archaeology and Heritage Science Facility. Twenty‐two were drawn from the early‐medieval Black Gate, Newcastle‐upon‐Tyne collection and 21 from the post‐medieval Coronation Street, South Shields collection. As simulation studies have suggested the imputation of > 10% missing data can alter statistical patterns derived from the covariance matrix, only relatively complete os coxae were selected, limiting sample size (Wigley and Blackwell 2025).

The excavation of the Black Gate cemetery in Newcastle‐upon‐Tyne took place between 1977 and 1992, leading to the recovery of 643 skeletons. The majority of the burials date from approximately the late‐7th century to 1080 ad, with a small number of burials made after that point (Mahoney Swales 2019; Nolan et al. 2010, 148). The individuals recovered from Black Gate likely lived in the rural environs of a pre‐industrial center and potentially experienced seasonal variability in resource availability (Mahoney Swales and Nystrom 2009; Mahoney Swales 2019, 202). The Coronation Street collection is associated with the church of St Hilda's, South Shields. The portion of the cemetery excavated between 2006 and 2007 ahead of construction work contains burials from the mid‐18th to mid‐19th centuries AD. In total, 204 individual skeletons were recovered. The population of Coronation Street inhabited an industrial town and were exposed to various pollutants, industrial hazards, and had limited access to fresh nutritional resources (Raynor et al. 2011, 76). In short, the populations represented by these skeletons lived in very different environments and experienced different stressors throughout development, facilitating the exploration of physiological stress on os coxae integration.

## 3 Methods

### 3.1 Osteological

Sex is associated with heterogeneity in frailty, with males typically less resilient to physiological stressors (e.g., DeWitte and Yaussy 2019; DeWitte 2018). To better explore associations between developmental integration and stress, skeletal sex was estimated as a proxy for biological sex. Skeletal sex was estimated with reference to dimorphic traits in the pelvis and skull (Table 1). Traits were graded along a 5‐point scale according to standard protocols (Buikstra and Ubelaker 1994, 15–20; Ferembach et al. 1980; White et al. 2012, 408–418). Scores were then averaged and simplified so that (1–2 = female; 0 = indeterminate; 3–5 = male).

**TABLE 1:** Traits utilized to estimate skeletal sex.

Pelvic traits	Cranial traits
– Ventral arc – Subpubic concavity – Ischiopubic ramus – Iliac crest curvature – Sciatic notch	– Nuchal crest – Mastoid process – Supraorbital margin – Glabella – Mental eminence – Gonial angle – Mandibular angle and flexure

Developmental stress experience was inferred through the presence or absence of chronologically matched LEH in the permanent dentition. All permanent teeth available were assessed and LEH were graded present when a trough‐like furrow could be observed crossing the surface of the tooth (Goodman and Rose 1990, 67; Guatelli‐Steinberg 2003, 311; Hillson 1996, 167–166; Kinaston et al. 2019, 753–756). The location of LEH was recorded with reference to the atlas of Primeau et al. (2015) which divides each tooth into bands which develop during an approximate one‐year age range between 1 and 12 years. It was thus possible to estimate the timing of defect formation. When two or more defects were observed in the same band on different teeth, it was taken as evidence of a physiological stress event (e.g., Temple et al. 2012, 1636). LEH that could not be matched were not deemed evidence of a stress event as they could be the result of localized trauma. Similarly, other enamel defects (e.g., pitting and hypocalcification) were not recorded due to their potential genetic etiology (Towle and Irish 2020). Associations between groups (i.e., site and sex) and the presence/absence of matched LEH were quantified through *χ* ^2^ tests. Due to the small sample sizes evaluated, E. Pearson's “*N* − 1” version of the χ^2^ test was employed; this method mitigates against the overestimation of the χ^2^ statistic in small samples, providing a better estimate of the true *p*‐value than the conventional test (Campbell 2007; Pearson 1947; Van Pool and Leonard 2010).

### 3.2 Scanning, Digitization, and Registration

Ossa coxae were scanned and digitized according to the method outlined in Wigley and Blackwell (2025). An Artec Eva structured‐light scanner was used to produce three‐dimensional scans (Artec 2023; Marić et al. 2022). Preferentially, the left side was scanned. When this was not possible, right‐side scans were reflected 180° about a vertical axis; this and similar approaches have been employed in studies of bilateral skeletal elements and are feasible due to the relatively small contribution made by directional and fluctuating asymmetry to overall morphological variation (e.g., Betti et al. 2013; Fischer and Mitteroecker 2017, 699). In Viewbox 4 (version 4.1.0.12, dHAL Software), scans were defined through a template of 107 coordinate points which have proven effective for decomposing statistical relationships between modules in the human os coxae (Wigley and Blackwell 2025) (see Tables S1 and S2). These coordinates reflected the location of homologous landmarks and semi‐landmarks which subsampled curves along the borders of the structure; coordinate points were labeled according to whether they were located on the ilium, ischium, or pubis (Standring 2016, 1337–1348; White et al. 2012, 227–240; Wigley and Blackwell 2025). This process summarized os coxae morphology through configurations of coordinate points which were comparable between individuals.

Following scanning and digitization, configurations were read into the R environment (R Core Team 2023). Once in R, the small amount of missing data (4.2% of points) lost due to taphonomic damage was filled. To do this, first all complete configurations were superimposed and registered in Kendall's shape space through a Procrustean procedure which utilized a least squares optimization algorithm to remove differences between individual shapes due to location, size, and orientation (Dryden and Mardia 2016, 126–136; Gower 1975). A consensus shape was computed from these configurations and the corresponding points from this shape were used to register incomplete configurations in shape space (Arbour and Brown 2014, 17–18; Arbour and Brown 2022). Random Forest imputation using the *missForest* algorithm was then employed to impute missing coordinate points (Stekhoven and Bühlmann 2012; Stekhoven 2022, 2–6). The *missForest* algorithm initially fills missing points with a synthetic value (e.g., the mean). Values for each absent point were successively imputed through a trained random forest composed of 100 “trees” constructed from observed data; configurations with the most missing data were handled before those with the least. This process was repeated 10 times and results averaged (Stekhoven and Bühlmann 2012; Stekhoven 2022, 2–6; Tang and Ishwaran 2017, 2–3; Wigley and Blackwell 2025, 130). Configurations were then re‐aligned and registered in the same shape space (Bookstein 1997; Gunz and Mitteroecker 2013; Perez et al. 2006, 770; Zelditch et al. 2012, 123). Simulation studies have shown this approach to be effective at preserving patterns of statistical covariation in damaged skeletal elements (Wigley and Blackwell 2025).

For inferential purposes, configurations were projected into tangent linear space where the Euclidean distances between configurations could be decomposed (i.e., through a Procrustes ANOVA procedure) to investigate factors affecting shape (Dryden and Mardia 2016, 88–95; Goodall 1991, 314; Klingenberg and McIntyre 1998). Centroid size, found as the square root of the sum of squared Euclidean distances of each landmark from the configuration's centroid, was used as a measure of size (Dryden and Mardia 2016, 31–57).

### 3.3 Integration

Sample‐level patterns of os coxae integration were quantified using standard procedures. Initially, the covariance matrix (S), was divided into blocks which summarized morphological relationships within and between the three os coxae modules (i.e., the ilium, ischium, and pubis). This is more easily illustrated in a structure with two modules, which would be separated into: blocks $S_{11}$ and $S_{22}$ containing the covariances among the coordinate points in the first and second modules; block $S_{12}$ containing the covariances of coordinates between the two modules; and block $S_{21}$ which is the transpose of the latter (Zelditch and Swiderski 2023, 149). Integration was quantified through the rPLS coefficient following the method outlined in Bookstein et al. (2003). In brief, a partial least squares analysis, based on a singular value decomposition, was applied to $S_{12}$ to extract the paired linear combinations which maximized the covariance between blocks. rPLS ranges between 0 and 1, with values closer to 1 representing higher correlations between modules and elevated integration (Adams and Collyer 2016, 2624; Rohlf and Corti 2000). Average pairwise coefficients were calculated between the os coxae modules and significance was determined through a permutation procedure. In this permutation procedure the coordinate points from a module were shuffled, disassociating covariation between modules, so that the coefficient could be iteratively recalculated to generate a distribution of possible outcomes under the null hypothesis which were used to determine significance (i.e., when the observed rPLS coefficient fell beyond the 95th percentile) (Adams and Collyer 2016, 2624; Zelditch and Swiderski 2023, 149).

The rPLS coefficient is dependent on sample size and number of points (Adams and Collyer 2016; Conaway and Adams 2022). While the number of coordinate points per configuration did not vary, the Leave‐One‐Out approach (LOO) (see below) employed to compute individual integration scores compared two samples of size n and n−1 respectively. As such, a standardized z score was computed which was not dependent on sample size. This was found as

$$z=\frac{\text{rPLS}-{\hat{\mu }}_{r}}{{\hat{\sigma }}_{r}}$$

where ${\hat{\mu }}_{r}$ and ${\hat{\sigma }}_{r}$ are the mean and standard deviation of the sampling distribution employed to estimate the expected value of rPLS under the null hypothesis (see above) (Adams and Collyer 2016, 2625; Zelditch and Swiderski 2023, 150).

To compute an individual estimate of developmental integration for the ith individual where i=1,…,n, a Leave‐One‐Out approach was taken. When this approach is taken, parameter estimates or a model are generated with a full dataset, and subsequently each individual data point is removed, and the estimates/model are re‐generated without that specific datum. This is repeated for each point/row in the dataset. The LOO approach has diverse applications in data science including, for example, cross‐validation (to assess model performance), sensitivity analysis, and outlier detection (Gelman et al. 2014, 1004–1005; George et al. 2015). In the latter case, the LOO technique functions to determine the impact of individual datum on global patterns (e.g., George et al. 2015). The LOO approach is employed similarly here, but instead of evaluating the impact of individual datum on parameter estimates/models, it is used to estimate of the contribution of the absent individual to group‐level patterns (e.g., Saggar et al. 2015, 276). As such, each configuration in the analysis was omitted and integration coefficients and z scores were recalculated. The z score from the LOO analysis was subtracted from the z score from the full analysis to produce $z_{i}$. Positive and higher valued $z_{i}$ scores indicate increased integration, while negative and lower scores indicate decreased integration. While the use of standardized z scores mitigate the sample size dependence discussed previously, individual $z_{i}$ scores are dependent on sample composition and are a relative measure, limiting cross‐sample comparability (discussed below).

A suite of standard univariate tests was employed to explore $z_{i}$ scores. These included a Kolmogorov–Smirnov test to ascertain whether scores approximated a normal distribution. Due to the presence of outliers and significant Kolmogorov–Smirnov test result (see below), non‐parametric Mann–Whitney *U* tests were employed to investigate differences between groups; Vargha and Delaney's *A* statistic, which provided the probability that a $z_{i}$ score from one group would be greater than from the other group, was computed as an effect size (Mann and Whitney 1947; Van Pool and Leonard 2010; Vargha and Delaney 2000).

## 4 Results

### 4.1 Osteological

Following sex estimation, 23 individuals were estimated to be female and 20 male. While the distribution was not equal between sites, with females (*n* = 14) better represented among the Black Gate skeletons than males (*n* = 8), and males (*n* = 12) more common in the Coronation Street sample than females (*n* = 9), there was no significant association between site and sex (χ^2^ = 1.821, *p* = 0.177).

Thirty individuals had surviving dentition. Among these, 19 had teeth with LEH that could be chronologically matched using the atlas of Primeau et al. (2015), and 11 were absent for matched LEH. There was a nearly equal number of individuals from Black Gate for whom matched LEH were present (*n* = 8) and absent (*n* = 9). Among the Coronation Street skeletons, matched LEH were more frequently present (*n* = 11) than absent (*n* = 2), and a significant association between site and matched LEH presence was detected (χ^2^ = 4.325, *p* = 0.0376). There was also a significant association between sex and matched LEH presence (χ^2^ = 6.799, *p* = 0.009). In females, absence (*n* = 9) of matched LEH was more frequent than presence (*n* = 6). For males, matched LEH presence was the norm (*n* = 13), rather than absence (*n* = 2).

### 4.2 Geometric Morphometric

Following preparatory procedures and registration of coordinate configurations in tangent linear space, integration was estimated at the sample level (rPLS=0.814, *p* = 0.001, z=5.681). Individual integration scores were then calculated through the Leave‐One‐Out approach; $z_{i}$ scores were distributed about a point of central tendency around approximately 0 (Table 2). A significant Kolmogorov–Smirnov test (*D* = 0.378, *p* = <0.001) suggested that $z_{i}$ scores did not approximate a normal distribution which, along with the presence of outliers in between‐group comparisons, necessitated the use of non‐parametric procedures in further testing.

**TABLE 2:** Summary of $z_{i}$ scores.

Score	Minima	1st Quartile	Median	Mean	3rd Quartile	Maxima	Standard deviation
$z_{i}$	−0.613	−0.024	0.221	0.205	0.438	0.854	0.317

**FIGURE 2:**
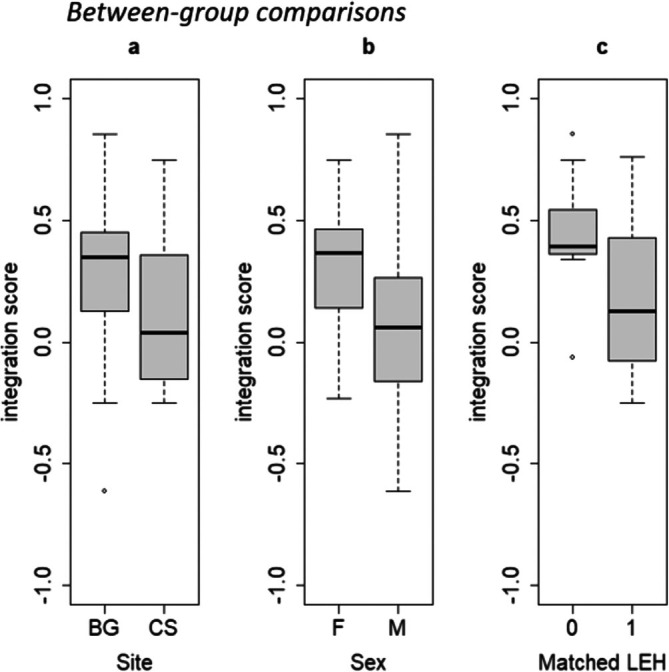
Boxplots contrasting developmental integration as implied by $z_{i}$ scores between the Black Gate (BG) and Coronation Street (CS) (a), females (F) and males (M) (b), and individuals for whom matched LEH were absent (0) and present (1) (c).

Mann–Whitney U tests revealed significant differences between groups in $z_{i}$ scores (Tables 3, 4, 5; Figure 2). A significant difference between sites with medium effect size was found (*U* = 319, *p* = 0.033, *A* = 0.690), with the Coronation Street sample having lower $z_{i}$ scores. There was a significant difference with medium effect size between sexes (*U* = 337, *p* = 0.009, *A* = 0.733); males had lower $z_{i}$ scores. A significant difference with large effect size was detected between those with and without matched LEH (*U* = 157, *p* = 0.024, *A* = 0.751); lower $z_{i}$ scores were found among the group with matched LEH.

**TABLE 3:** $z_{i}$ scores compared between sites.

Site	No.	Minima	1st Quartile	Median	Mean	3rd Quartile	Maxima	Standard deviation
Black Gate	22	−0.613	0.149	0.350	0.294	0.452	0.854	0.320
Coronation Street	21	−0.252	−0.153	0.039	0.111	0.358	0.750	0.293

**TABLE 4:** $z_{i}$ scores compared between sexes.

Sex	No.	Minima	1st Quartile	Median	Mean	3rd Quartile	Maxima	Standard deviation
Females	23	−0.231	0.140	0.366	0.314	0.463	0.750	0.239
Males	20	−0.613	−0.157	0.062	0.080	0.242	0.854	0.354

**TABLE 5:** $z_{i}$ scores compared between matched LEH absent/present groups.

Matched LEH	No.	Minima	1st Quartile	Median	Mean	3rd Quartile	Maxima	Standard deviation
Absent	11	−0.0661	0.364	0.395	0.446	0.543	0.854	0.241
Present	19	−0.252	−0.079	0.125	0.166	0.429	0.762	0.307

A Procrustes ANOVA revealed that $z_{i}$ scores did not predict shape. Shape was better explained by sex (*p* = 0.001) and site (*p* = 0.011) (Table 6). A Spearman's correlation test found there was no significant correlation between $z_{i}$ scores and os coxae size (*ρ* = −0.07, *p* = 0.670).

**TABLE 6:** A Procrustes ANOVA procedure identified factors affecting os coxae morphology. Significance was determined through a Randomized Residual Permutation Procedure with 1000 permutations (Adams et al. 2023).

Effects	df	SS	MS	*R* ^2^	*F*	*p*
Sex	1	0.024	0.024	0.096	4.570	0.001
Site	1	0.010	0.010	0.039	1.800	0.011
Residuals	40	0.210	0.005	0.863		
Total	42	0.244				

## 5 Discussion

### 5.1 Integration and Stress

A significant difference with large effect size was observed between individuals with and without matched LEH (*U* = 157, *p* = 0.024, *A* = 0.751), with individuals that exhibited matched LEH generally having lower $z_{i}$ scores. Due to the low statistical power and consequent increased risk of Type I error when dealing with small samples (here only 30 individuals could be assessed for matched LEH presence/absence), inferences drawn from this result must be treated cautiously. With this in mind, the effect size suggests that there is a 0.751 probability that individuals without matched LEH will have a higher integration score than an individual with matched LEH (Vargha and Delaney 2000). LEH are a marker of stress during development, with factors such as resource scarcity (e.g., undernutrition) and experiences which cause an increased demand for physiological capital (e.g., disease experience) being implicated in development (Guatelli‐Steinberg 2003, 311; Hillson 1996, 167–166; Kinaston et al. 2019, 753–756). This paper therefore presents findings which associate developmental integration and physiological stress.

Associations were also found between integration scores and groups with differing exposures or vulnerabilities to stress. A significant difference with medium effect size was detected between sites (*U* = 319, *p* = 0.033, *A* = 0.690). Coronation Street was generally associated with lower scores, and tests imply there is a 0.690 probability of individuals from Black Gate exhibiting higher $z_{i}$ scores (Vargha and Delaney 2000). Although to the authors' knowledge there are no published direct comparisons of the two sites, it is plausible that the population of post‐medieval South Shields, from which the Coronation Street sample is derived, experienced substantial physiological stress. South Shields was home to various industries, including collieries, chemical works, and gas works (Raynor et al. 2011, 106). Even by contemporary standards the environment was considered highly populated, hazardous, and detrimental to health (e.g., Report of the Commissioners 1845, 185). Modern osteological comparisons between post‐medieval assemblages support the supposition that South Shield's population was subject to stressors which impacted morbidity and mortality risk (Raynor et al. 2011). A significant difference was furthermore observed between sexes (*U* = 337, *p* = 0.009, *A* = 0.733), with males having on average lower $z_{i}$ scores. This aligns with well‐established differentials in frailty and again links reduced developmental integration in skeletal structures to elevated physiological stress experience. It has, for example, been found that during the Black Death (i.e., a time in which populations were exposed to severe physiological stress), males developed more skeletal stress markers, reduced skeletal growth, and higher mortality risk (DeWitte 2018). While this patterning of life course outcomes could represent male survival, it also supports the argument that males are more vulnerable to stress and this can manifest in the skeleton (DeWitte and Stojanowski 2015; Wood et al. 1992).

The alternative hypothesis articulated at the beginning of this paper proposed that developmental integration would be lower in individuals with evidence of elevated physiological stress during development (i.e., LEH) or in groups more susceptible to stress. The data are consistent with this hypothesis and thus it might be plausible that variation in $z_{i}$ scores is associated with developmental stress experience. Possibly elevated stress subtly disrupts normal os coxae growth, reducing morphological integration. This interpretation must be treated carefully, however. Given the observational nature of this study, causation cannot be implied. The significant association of matched LEH with site (χ^2^ = 4.325, *p* = 0.0376) and sex (χ^2^ = 6.799, *p* = 0.009) also problematizes the supposition that individuals from the Coronation Street sample and males had lower $z_{i}$ scores, and therefore reduced os coxae integration, due to elevated stress exposure and heterogenous frailty. It is likely that site‐specific stressors and sex differentials in resilience did not operate independently and rather interacted to influence stress experience and the prevalence of matched LEH. Unfortunately, due to small sample size, it was not possible to investigated relationships between factors in more detail with multivariate statistical procedures. Moreover, while skeletal stress markers such as LEH do reflect developmental stress experience, they also indicate survival of that stress while the absence of markers could either reflect the absence of stress or that the individual in question did not survive stressors long enough to develop markers (i.e., Osteological Paradox) (DeWitte and Stojanowski 2015; Wood et al. 1992). This interpretative paradox prevents clear links being drawn between stress experience and skeletal stress markers.

The concept that developmental integration is linked to variation in stress experience may add a new facet to past research, however, which has largely focused upon the genetic underpinnings and evolutionary significance of integration (e.g., Bookstein et al. 2003; Cowley and Atchley 1990; Hlusko and Mahaney 2009; Willmore et al. 2009), rather than the impact of factors experienced over an individual's life. Parallels can be found in previous work. For instance, the supposition that the growth of adjacent developmental modules can be influenced by physiological demands across “competing” modules is supported by experimental protocols in the plant and animal sciences which have observed abnormal and increased localized growth when the development of neighboring modules has been perturbed (Klingenberg and Nijhout 1998, 1135–1136; Van Dongen et al. 1999, 87–88). Rivera and Deppen Neely (2020) also found that stress‐induced departures from symmetry in the limb bones of vertebrates were generally slight and smallest in limbs under greatest load during locomotion, indicating that skeletal morphology is highly conserved in functionally significant elements and that deviations from target phenotype tend to be small and stress‐induced. Given its key role in locomotion, thermoregulation, and childbirth (Candelas Gonzalez et al. 2017; Kurki 2007; Ruff 1993; Standring 2016, 1337–1348; Warrener 2023), it is expected that os coxae morphology is similarly conserved and that stress‐induced variation is small, except in the most severe circumstances. This is consistent with the finding that $z_{i}$ scores could not be linked to variation in os coxae shape or size, suggesting that they represent minor variations in morphology.

Given the association with developmental stress markers revealed here, measures of integration may have potential as a skeletal stress marker in much the same way as fluctuating asymmetry and hypoplastic defects (e.g., Guatelli‐Steinberg 2003; Wigley et al. 2024). If so, this method could be extended to other skeletal elements with clearly defined modules. Utilized in this manner, individual measures of integration could represent an attractive addition to the suite of stress markers available to biological anthropologists and bioarcheologists, though it is recommended that they are used in conjunction with other stress markers so that normal and stress‐induced variation are not conflated (i.e., otherwise in relatively homogenous samples, small deviations could be perceived as associated with stress). More work will also be needed to address the limitations discussed below.

### 5.2 Limitations and Future Research

This paper has presented a novel combination of existing methods through which it is possible to calculate individual scores reflecting variation in developmental integration (i.e., $z_{i}$ scores), enabling multi‐scale investigations. By quantifying patterns of trait correlation in this way it is possible to explore within‐group differences and test hypotheses. The limitations in the study presented here and discussed below do, however, imply that further work is needed to validate the approach.

The use of matched LEH as a stress marker here presents several complications. In this project, LEH were matched across zones of the dentition and, when multiple LEH were observed in synchronously forming zones, employed to imply an individual had experienced an episode of stress during development (Primeau et al. 2015; Temple et al. 2012). This method helps to mitigate against recording LEH with etiologies other than stress (e.g., genetic or traumatic) (Towle and Irish 2020). However, the imbricational enamel of later‐forming teeth has mineralized by approximately the twelfth postnatal year (Primeau et al. 2015; Reid and Dean 2006, 334). In contrast, the os coxae follows a different developmental tempo. Depending on factors such as sex, stress experience, and pubertal development, certain parts of the os coxae (e.g., the iliac crest) may not reach their mature form until past the twentieth year (Lewis et al. 2016; Schaefer et al. 2009, 229–254). Thus, while LEH may preserve an archaeologically durable record of stress due to the resistance of enamel to taphonomic factors, here they cannot capture stress over the whole period that os coxae are developing; times of elevated developmental plasticity (i.e., sensitive developmental windows) will also differ (McPherson 2021; Schaefer et al. 2009; White et al. 2012). In addition to this, os coxae and teeth will be affected differently by stressors as the processes underlying the development of bone and enamel are different (Reid and Dean 2006; Schaefer et al. 2009; Hillson 1996). In short, the proposed association between os coxae integration and developmental stress as implied through matched LEH warrants further validation, possibly incorporating a broader range of stress markers.

The impact of biomechanically stimulated remodeling with age, size differences, and secular changes should be considered as complicating factors. Each module of the os coxae acts as a point of origin and insertion for muscles that contribute variably to differing movements. It is not unreasonable to assume that remodeling in response to loading throughout life could cause localized and age‐dependent alterations to morphology which may impact estimates of integration (Gosling et al. 2002; Hallgrímsson 1999, 145; Standring 2016, 1337–1348; White et al. 2012, 227–240). Allometric affects, or the tendency for growth and therefore shape to vary as structures become larger, should also be taken into account if the method is applied (Klingenberg 2015, 896). It is not believed this was a confounding factor here, however, as there was no discernible correlation between $z_{i}$ scores and size (ρ = −0.07, *p* = 0.670). In addition, when comparing sites, it has been supposed that developmental variation of the os coxae in modern humans has not altered substantially over time. While to the authors' knowledge there is currently no literature to suggest it has, genetic drift, selective pressures, and epigenetic processes cause directional changes in development (e.g., stature, pubertal tempo) (e.g., Lewis et al. 2025), and could feasibly alter patterns of developmental integration and complicate comparisons. Given the relatively small sample size drawn from the Black Gate and Coronation Street collections (Nolan et al. 2010; Raynor et al. 2011), the representativeness of the samples must also be queried.

The biological meaningfulness of $z_{i}$ scores should be considered. As scores did not correlate with centroid size, and os coxae shape was better explained by sex and site, it is not likely that variation in $z_{i}$ scores is indicative of morphological changes which impact function. Past research has yielded divergent explanations of the factors responsible for os coxae form. Some research suggests that neutral selective processes (i.e., genetic drift) operating in spatiotemporally distant populations are responsible for global idiosyncrasies in form (e.g., Betti et al. 2013). Other work highlights the varied aspects of human function influenced by os coxae size and shape (e.g., childbirth, bipedalism, thermoregulation) (Candelas Gonzalez et al. 2017; Kurki 2007; Ruff 1993; Warrener 2023). It has been proposed that the competing demands of these functional considerations impose selective pressures, with the adaptive significance of traits varying between physical and cultural environments, thereby contributing to population‐specific differences (Mitteroecker et al. 2021). As a metric derived from morphological patterns, it could be argued that $z_{i}$ scores represent variation associated with either neutral or selective factors rather than stress. This, however, seems unlikely given the significantly lower scores detected among individuals with matched LEH. Unlike LEH, however, $z_{i}$ scores summarize minor trait variations that are only statistically identifiable and, being a relative measure, will vary according to sample composition. Potentially, therefore, the approach articulated here holds limited appeal when compared to traditional osteological stress markers (e.g., LEH) which are clearly visible and reflect obvious pathological changes. However, a similar critique could be made of the analysis of fluctuating asymmetry which is again a summary statistic dependent on sample composition and reflective of small, non‐pathological phenotypic variations without functional consequence. Analyses of fluctuating asymmetry have been a useful interrogative tool in diverse fields (e.g., Klingenberg 2015; Radwan et al. 2003; Weisensee 2013), but not an uncomplicated one, with meta‐analyses struggling to detect consistent substantial links with important outcomes such as morbidity, fecundity, and mortality risk (e.g., Leung and Forbes 1996; Møller 1999). As such, the utility of $z_{i}$ scores as a biological marker associated with stress needs to be established through work which encompasses meaningful life course outcomes (e.g., growth profiles, age‐at‐death estimation) in larger samples. This may help to ascertain what magnitude of variation in developmental integration represents a deviation from normal with‐group variation.

Future work may also consider models other than the three‐module model presented here. As it was the aim of the paper to explore associations between physiological stress experienced during development and integration, the selection of a three‐module model seemed logical given the ilium, ischium, and pubis initially develop as three separate bones. However, functionally it could be better to consider the os coxae as two modules, specifically the “true pelvis” and “false pelvis” (Kurki 2007, 1152; Warrener 2023). The iliopectineal line divides these two regions of the pelvis. The true pelvis is largely composed of the pubis and ischium, and it has been proposed its size and shape are influenced by obstetric and locomotor demands. Meanwhile, the false pelvis encompasses much of the ilium, and it has been posited that its form is impacted by thermoregulatory requirements linked to environmental conditions (Betti 2014, 168; Ruff 1993). Again, however, this simplifies the functional role, life history, and evolutionary history of the os coxae which has evolved under the influence of varied functional, environmental, and genetic factors (e.g., Betti 2014; Warrener 2023). Any model devised will likely have shortcomings, so must be adapted to test specific hypotheses. Regarding further directions of investigation, it may be possible to apply the approach described here to measures of modularity, which are found by assessing trait dependence between modules (rather than independence) (Adams 2016; Zelditch and Swiderski 2023). However, when attempted with this dataset, while expected inverse patterns were observed (i.e., groups that had lower integration had higher modularity), only differences between those with and without matched LEH were significant.

## 6 Conclusion

This paper has presented a novel combination of existing methods to summarize the extent to which a skeletal structure's morphological traits are correlated. Utilizing geometric morphometric techniques and a Leave‐One‐Out approach an individual score was computed expressing the magnitude of developmental integration in relation to group‐level trends, leading to the potential for multi‐scale investigations (Adams 2016; Adams et al. 2023; Bookstein et al. 2003; Saggar et al. 2015, 276). Integration scores were compared between sites, sexes, and individuals with and without markers of physiological stress experienced during development (i.e., matched LEH). Statistical exploration revealed a significant difference with larger effect size in integration scores between those with and without LEH, with lower integration scores among the group with these dental defects. Significantly lower integration scores were also found among groups known to have inhabited an environment harmful to health (i.e., the Coronation Street sample from industrial South Shields), or be more vulnerable to stress due to differentials in frailty (i.e., males) (DeWitte and Yaussy 2019; DeWitte 2018; Raynor et al. 2011). It is therefore speculated that the method described here can be adapted to explore stress experience in skeletal samples, but that further work with larger samples is needed to validate this assumption and the association of variation in developmental integration with physiological stress experience.

## Author Contributions

**B. R. Wigley:** conceptualization, investigation, funding acquisition, writing – original draft, methodology, visualization, writing – review and editing, formal analysis, project administration, data curation. **P. G. Blackwell:** investigation, funding acquisition, writing – review and editing, formal analysis, project administration, supervision, validation, methodology.

## Funding

This work was supported by the Engineering and Physical Sciences Research Council (EPSRC). For the purpose of open access, the author has applied a Creative Commons Attribution (CC BY) license to any Author Accepted Manuscript version arising.

## Ethics Statement

Permission to access human skeletal collections for non‐destructive research purposes was provided by the University of Sheffield's Department of Archaeology.

## Conflicts of Interest

The authors declare no conflicts of interest.

## Supporting information


**Data S1:** Supporting information.

**Table S1:** Locations of landmarks. Landmarks were categorized as anatomical (“anat”), mathematical (“math”) or pseudo following Dryden and Mardia (2016, 3–5). Conjugate landmarks refer to those located at points where two or three of the pelvic bones fuse.
**Table S2:** The curves employed to define ossa coxae, the points used to anchor the start and end of each curve, and the density of semi‐landmark points located on each curve. Regarding the latter, curves with a low density of semi‐landmarks had three points initially placed at intervals every 25% of the curve's length. Moderate and high density curves had four and nine semi‐landmark points initially placed at intervals of 20% and 10% respectively. Homology between semi‐landmark points was attained after handling to minimize bending energy (Bookstein 1997; Gunz and Mitteroecker 2013; Perez et al. 2006, 770). Refer to Wigley and Blackwell (2025) for further details on how the density and placement of landmarks was determined.

## Data Availability

The data analyzed in this paper is stored in INDIGO, the University of Illinois Chicago's online data repository and available at https://indigo.uic.edu/articles/dataset/Data_Developmental_integration_and_physiological_stress/32675718.
