# Supplementary material for: Investigating Associations Between Developmental Integration and Physiological Stress
Source: Am J Biol Anthropol. 2026 Jun 23;190(2):e70297. doi: 10.1002/ajpa.70297 (PMC13290501; doi:10.1002/ajpa.70297)
Supplement: Supplementary file 2 — Table S1: Locations of landmarks. Landmarks were categorized as anatomical (“anat”), mathematical (“math”) or pseudo following Dryden and Mardia (2016, 3–5). Conjugate landmarks refer to those located at points where two or three of the pelvic bones fuse. Table S2: The curves employed to define ossa coxae, the points used to anchor the start and end of each curve, and the density of semi‐landmark points located on each curve. Regarding the latter, curves with a low density of semi‐landmarks had three points initially placed at intervals every 25% of the curve's length. Moderate and high density curves had four and nine semi‐landmark points initially placed at intervals of 20% and 10% respectively. Homology between semi‐landmark points was attained after handling to minimize bending energy (Bookstein 1997; Gunz and Mitteroecker 2013; Perez et al. 2006, 770). Refer to Wigley and Blackwell (2025) for further details on how the density and placement of landmarks was determined. [file AJPA-190-e70297-s002.docx]

| **Element** | **Point** | **Landmark Description** | **Type** |
| --- | --- | --- | --- |
| Ilium | 1 | Union between the base of the anterior inferior iliac spine and the margin of the acetabulum | anat |
|  | 2 | Most prominent point of the anterior inferior iliac spine (AIIS) | math |
|  | 3 | The base of the anterior superior iliac spine (ASIS) | math |
|  | 4 | Midpoint of the supero-lateral edge of the iliac tubercle | math |
|  | 5 | Most prominent point of the posterior superior iliac spine (PSIS) | math |
|  | 6 | Most prominent point of the posterior inferior iliac spine (PIIS) | math |
|  | 7 | Most superior point on the auricular surface of the ilium | math |
|  | 8 | The point where the arcuate line meets the auricular surface | anat |
|  | 9 | Deepest point of the sciatic notch | math |
| Ischium | 10 | Tip of the ischial spine | math |
|  | 11 | Apex of the ischial tuberosity | math |
|  | 12 | Most inferior point of the ischial tuberosity | math |
|  | 13 | Most anterior point of the inferior end of the acetabular lunate surface | math |
| Pubis | 14 | Most inferior point of the pubic symphysis | math |
|  | 15 | Most superior point of the pubic symphysis | math |
|  | 16 | Apex of the pubic spine | math |
|  | 17 | Most anterior point of the superior end of the acetabular lunate surface | math |
| Conjugate | 18 | The acetabular point – i.e., the anterior angle of the superior acetabular lobe, following Rissech *et al*. (2001) | anat |
|  | 19 | Iliopectineal eminence | anat |
|  | 20 | Point on the lateral aspect of the ridge of the obturator foramen located at the thickened position where the pubic and ischial rami fuse | anat |
|  | 21 | Point on the medial aspect of the ischiopubic ramus located at a right angle to landmark 20 | pseudo |
|  | 22 | Point on the obturator foramen located on a line projecting from landmark 18 and intersecting midway the line between landmarks 14 and 17 | pseudo |
|  | 23 | Halfway between the point of maximum curvature in the greater sciatic notch (lm 9) and the ischial spine (lm 10) | math |

Supplementary Table 1 Locations of landmarks. Landmarks were categorised as anatomical (“anat”), mathematical (“math”) or pseudo following Dryden and Mardia (2016: 3-5). Conjugate landmarks refer to those located at points where two or three of the pelvic bones fuse.

| **Element** | **Curve** | **Starting Point** | **Final Point** | **Semi-landmark Density** |
| --- | --- | --- | --- | --- |
| Ilium | 1 | 1 | 2 | Low |
|  | 2 | 2 | 3 | Low |
|  | 3 | 3 | 4 | Moderate |
|  | 4 | 4 | 5 | High |
|  | 5 | 5 | 6 | Low |
|  | 6 | 6 | 9 | Low |
|  | 7 | 9 | 23 | Low |
|  | 8 | 19 | 8 | Moderate |
|  | 9 | 7 | 8 | Low |
|  | 10 | 8 | 6 | Low |
|  | 11 | 7 | 6 | Moderate |
| Ischium | 12 | 23 | 10 | Low |
|  | 13 | 10 | 11 | Low |
|  | 14 | 11 | 12 | Moderate |
|  | 15 | 11 | 12 | Moderate |
|  | 16 | 12 | 21 | Moderate |
|  | 17 | 22 | 20 | Moderate |
| Pubis | 18 | 21 | 15 | Low |
|  | 19 | 15 | 16 | Low |
|  | 20 | 16 | 19 | Moderate |
|  | 21 | 22 | 20 | Moderate |
|  | 22 | 15 | 14 | Low |
|  | 23 | 15 | 14 | Low |

Supplementary Table 2 The curves employed to define ossa coxae, the points used to anchor the start and end of each curve, and the density of semi-landmark points located on each curve. Regarding the latter, curves with a low density of semi-landmarks had three points initially placed at intervals every 25% of the curve’s length. Moderate and high density curves had four and nine semi-landmark points initially placed at intervals of 20% and 10% respectively. Homology between semi-landmark points was attained after handling to minimise bending energy (Bookstein 1997; Gunz and Mitteroecker 2013; Perez *et al*. 2006: 770). Refer to Wigley and Blackwell (2025) for further details on how the density and placement of landmarks was determined.

**BIBLIOGRAPHY**

Bookstein, F. L. 1997. Landmark methods for forms without landmarks: localizing group differences in outline shape. *Medical Image Analysis* 1: 225-243.

Dryden, I. L. and Mardia, K. V. 2016. *Statistical shape analysis, with applications in R: Second Edition*. Chichester: Wiley.

Gunz, P. and Mitteroecker, P. 2013. Semilandmarks: a method for quantifying curves and surface. *Hystrix* 24 (1): 103-109.

Perez, S. I., Bernal, V. and Gonzalez, P. N. 2006. Differences between sliding semi-landmark methods in geometric morphometrics, with an application to human craniofacial and dental variation. *Journal of Anatomy* 208: 769-784.

Rissech, C., Sañudo J. R. and Malgosa A. 2001. Short Report. The acetabular point: a morphological and ontogenetic study. *Journal of Anatomy* 198: 743-748.

Wigley, B. R. and Blackwell, P. G. 2025. Getting to the Point: Defining, Reconstructing and Investigating Shape Through a Procrustean Protocol. *Journal of Computer Applications in Archaeology* 8 (1): 125-138.
